# Editorial: Animal models of multiple sclerosis: can they advance future therapies?

**DOI:** 10.3389/fnmol.2023.1229625

**Published:** 2023-06-13

**Authors:** Tara Maria DeSilva, Olaf Stuve

**Affiliations:** ^1^Department of Neurosciences, Cleveland Clinic, Cleveland, OH, United States; ^2^Department of Neurology, University of Texas Southwestern Medical Center, Peter O'Donnell Brain Institute, Neurology Section, VA North Texas Health Care System, Dallas, TX, United States

**Keywords:** multiple sclerosis, animal models, neurodegeneration, demyelination, remyelination, neuroinflammation, autoimmunity

Current treatment strategies for multiple sclerosis (MS) are immunomodulatory, either preventing activation, proliferation, or extravasation of immune cells into the central nervous system (CNS). These treatments reduce the number of relapses and improve quality of life early in the disease course. However, neurodegeneration and permanent disability ultimately still give rise to progressive neurological decline despite pharmacotherapies in many patients. Mechanisms driving neurodegeneration are necessary for the advancement of pharmacological strategies and while animal models have served well in addressing inflammatory responses in MS it is unclear how they will advance therapies for progressive disease. On April 29 2022, the American Committee for Treatment and Research in Multiple Sclerosis (ACTRIMS) Young Investigator Summit organized an expert workshop titled “Animal Models of MS” to discuss many of the controversial issues regarding experimental models in the assessment of CNS autoimmune disorders. This current edition of Frontiers in Molecular Neurosciences is a synthesis of some of the scientific dialog.

The goal of this Research Topic is to explore how various animal models represent different mechanisms of MS disease pathology as well as their limitations. Understanding the advantages and disadvantages of animal models in the study of MS in relationship to unmet needs is intended to provide a thought-provoking discussion on the future of MS research. This collection of research articles surveys the interplay of innate and adaptive immune responses driving relapsing-remitting vs. progressive neurodegeneration as well as their role in driving demyelination and remyelination. These data are then considered in the context of pharmacological therapies and relevance for biomarker development in monitoring disease progression.

One of the predominant preclinical models used to study MS is experimental autoimmune encephalomyelitis (EAE), an adaptive immune-mediated model initiated by CNS-infiltrating myelin-reactive T cells. Although EAE is not a model of progressive disability, it does exhibit demyelination and axonal injury characterized in active lesions from human postmortem MS brains. The review by Voskuhl and MacKenzie-Graham provides an in-depth assessment of not only white matter pathology in EAE, but also gray matter pathology including neuronal cell body and synaptic loss and how these findings compare to human postmortem MS brains. The authors also address gray matter atrophy in EAE including the hippocampus, thalamus, and cerebral cortex which is not only observed in MS brains, but significantly correlates with clinical disability. In particular, thalamic atrophy is one of the strongest correlates of clinical disability in MS, which associates with damage in extra-thalamic regions as opposed to lesions directly in the thalamus, emphasizing the relevance of studying its afferent and efferent connections to understand mechanisms of neurodegeneration. The review by Mey and DeSilva summarizes how damage to the retino-thalamic pathway is found in MS regardless of a history of optic neuritis and the correlation of structural and functional assessments in the retina with neurodegenerative processes as well as clinical disability. Furthermore, these findings are also documented in the EAE model suggesting that the layer specific organization of the retina and its synaptic connectivity in the thalamus provide an important pre-clinical platform for the exploration of neuroprotective strategies.

Another important mechanism driving neurodegeneration is failure of remyelination, which not only leaves axons vulnerable to insult, but deprives them of essential metabolic support facilitated by healthy myelin. While toxin-based models of demyelination have been traditionally used to study remyelinating therapies, Gharagozloo et al. present compelling information that combinatorial models including immune-mediated pathology are necessary to understand the inflammatory environment in MS lesions that are hypothesized to inhibit remyelination. Packer et al. provide further insight into how oligodendrocytes alter the capacity of myelin regeneration in a model and age-dependent manner, which complicates pre-clinical testing of therapeutic strategies for MS. This review also provides a comprehensive understanding of remyelinating drugs used in MS clinical trials and Mey and DeSilva describes visual assessments used as part of the clinical outcome measures.

While visual measurements provide a strategy to synergize assessments in MS with preclinical models, the review by Wu argues that the cerebral spinal fluid (CSF) in animal models of MS is a relatively understudied specimen given its importance as a diagnostic criteria for MS. The cerebral spinal fluid (CSF) bathes the central nervous system. Immune cells located in brain border regions including the meninges and the choroid plexus release factors into the CSF that modulate brain function during both physiological and pathological states. In this issue, Wu outlines methodological approaches for the study of the CSF in diverse animal models including myeloid subsets and antigen receptor single-cell transcriptomics to further our understanding of neurodegenerative mechanisms in MS. Wu also provides the logical hypothesis that understanding immune responses in the CSF will reveal novel mechanisms of inflammation in parenchymal tissue adjacent to compartments that house the CSF including brain border regions. One such mechanism that has come to light in evaluating the CSF is the complement system. These findings have also been reflected in histopathological studies in human post-mortem MS brain as well as animal models demonstrating complement activation in microglial phagocytosis of myelin debris as well as synaptic loss. In their review, Saez-Calveras et al. discuss the history and ongoing observations of the biological impact of complement in CNS autoimmunity. They also explore the contribution of complement activation to both innate and adaptive immunity in EAE and MS and their potential relevance for future therapeutic interventions in progressive MS.

In line with the idea that it is critical to interrogate similar tissues and fluids in animal models and human MS patients to understand pertinent mechanisms regulating neuroinflammation, the gut-brain axis must certainly be considered. More recently, changes in the gut microbiome composition as well as gut dysbiosis have been the focus of intense investigation. In this Research Topic of Frontiers of Molecular Neurosciences, Melamed et al. discuss how investigations in the EAE model have contributed to our understanding of the role of gut microbiome in neuroinflammation and CNS autoimmunity. Interestingly, they discuss how common dietary modifiers, including caloric restriction, alcohol, probiotics, antibiotics, and fecal microbiome transplant regulate CNS inflammatory activity. The authors contend that novel interventions targeting the microbiome may alter the inflammatory profiles contributing to neurodegeneration in MS.

Lastly, an important consideration in modeling neurodegeneration in MS is the viral-induced hypothesis of MS. The strongest genetic factor associated with MS is the human leukocyte antigen HLA DRB1^*^15:01, a co-receptor for Epstein-Barr virus. Recent studies have highlighted a strong association between Epstein-Barr virus (EBC) infections and MS. Theiler's murine encephalomyelitis virus (TMEV) infection, unlike EAE, is a model of secondary autoimmunity in response to a pathogen as well as progressive disease. In this Research Topic, Pike et al. outline our current understanding of TMEV-associated CNS autoimmunity, including initiation of autoimmune responses subsequent to damage of TMEV-infected oligodendrocytes, bystander activation of myeloid cells and amplification of CNS inflammation, epitope spreading from viral dominant determinants to self CNS antigenic determinants, and molecular mimicry between TMEV and autoantigens within the brain and spinal cord. The authors speculate that TMEV-induced demyelinating disease (TMEV-IDD) will play a critical role in testing future MS therapeutics for progressive disease.

Overall, this Research Topic examines the myriad of animal models used to study specific disease processes in MS with novel insights for the future of therapeutic strategies to address neurodegeneration.

## Author contributions

TD and OS developed the concept for this Research Topic and drafted and revised the editorial. All authors contributed to the article and approved the submitted version.

